# Enhanced outdoor visual localization using Py-Net voting segmentation approach

**DOI:** 10.3389/frobt.2024.1469588

**Published:** 2024-10-09

**Authors:** Jing Wang, Cheng Guo, Shaoyi Hu, Yibo Wang, Xuhui Fan

**Affiliations:** College of Communication and Information Engineering, Xi’an University of Science and Technology, Xi’ an, China

**Keywords:** camera relocalization, coordinate attention, pyramidal convolution, landmark segmentation map, landmark voting map

## Abstract

Camera relocalization determines the position and orientation of a camera in a 3D space. Althouh methods based on scene coordinate regression yield highly accurate results in indoor scenes, they exhibit poor performance in outdoor scenarios due to their large scale and increased complexity. A visual localization method, Py-Net, is therefore proposed herein. Py-Net is based on voting segmentation and comprises a main encoder containing Py-layer and two branch decoders. The Py-layer comprises pyramid convolution and 1 × 1 convolution kernels for feature extraction across multiple levels, with fewer parameters to enhance the model’s ability to extract scene information. Coordinate attention was added at the end of the encoder for feature correction, which improved the model robustness to interference. To prevent the feature loss caused by repetitive structures and low-texture images in the scene, deep over-parameterized convolution modules were incorporated into the seg and vote decoders. Landmark segmentation and voting maps were used to establish the relation between images and landmarks in 3D space, reducing anomalies and achieving high precision with a small number of landmarks. The experimental results show that, in multiple outdoor scenes, Py-Net achieves lower distance and angle errors compared to existing methods. Additionally, compared to VS-Net, which also uses a voting segmentation structure, Py-Net reduces the number of parameters by 31.85% and decreases the model size from 236MB to 170 MB.

## 1 Introduction

Camera relocalization is a fundamental problem in computer vision tasks. It aims to infer a camera’s translation vector and rotation angle in the world coordinate system from RGB images, determining the camera’s precise position and orientation in a scene.Camera relocalization is the core of simultaneous localization and mapping as well as a key module in technologies such as virtual reality, augmented reality, and autonomous driving (Chen et al., 2021). Traditional camera relocalization methods often employ structure-from-motion (SFM) techniques to achieve high-precision camera localization by preserving the geometric information of a scene using three-dimensional (3D) point clouds (Shavit and Ferens, 2019). However, these methods use feature point matching that causes relocalization failure when dealing with complex scenes. Absolute pose estimation using deep learning (Kendall A. et al., 2015) overcomes the limitations of large memory occupancy and hardware of traditional methods. Scene coordinate regression (SCR)-based (Li et al., 2018) camera relocalization is a deep learning method and regresses two-dimensional (2D) image pixels to obtain a relation between the 2D pixels and 3D scene coordinates. Further, it uses a random sampling consistency (RANSAC) algorithm to select the best poses, considerably improving the relocalization accuracy. Additionally, image retrieval–based camera relocalization methods require matching the query image with an image database to find the most similar image (Balntas et al., 2018; Arandjelovic et al., 2016) for calculating the relative position of a camera (Laskar et al., 2017). These methods yield good relocalization results in the absence of presented scenes but have low localization speed.

SCR-based camera relocalization methods perform well in indoor scenes. Networks with multiple viewpoint constraints converge more easily than single-viewpoint models. Cai et al. (Cai and Zhan, 2019) enhanced SCR networks using geometric constraints and self-supervision, allowing the networks to learn reliable 2D to 3D relations and improving the training efficiency. Hierarchical scene coordinate networks offer better performance. Li et al. (Li et al., 2020) proposed HSCNet, which accurately predicts pixel scene coordinates from a single RGB image through multiple output layers, with the final layer predicting the 3D coordinates. Yang et al. (Yang et al., 2019) proposed SANet, which decouples model parameters from the scene using hierarchical coding, enabling the localization of unknown scenes and estimation of camera poses.

Outdoor scene relocalization faces many challenges, such as differences between datasets and real environments and varying scene properties, which affect relocalization accuracy. In the presence of duplicate structures in a scene, an uncertainty is generated in the positional solution. Duong et al. (Duong et al., 2020) proposed an efficient multioutput scene coordinate (EMOSC) method that combines machine learning and geometric methods. It is a multioutput depth forest regression method based on sparse feature detection, which greatly reduces the algorithm running time and improves the prediction accuracy. Wald et al. (Wald et al., 2020) introduced RIO-10 and a new metric: dense correspondence reprojection error. Dong et al. (Dong et al., 2021) developed an outlier-aware neural tree for high-precision camera relocalization in dynamic indoor settings, featuring decision trees, neural routes, and dynamic point filtering. SCR-based methods balance accuracy and computation time. Turkoglu et al. (Turkoglu et al., 2021) combined graph neural networks with image retrieval using relative positional loss for training. Bui et al. (Bui et al., 2022) proposed a simpler SCR algorithm using perceptrons and sparse descriptors, resulting in a smaller model. PixLoc (Sarlin P. et al., 2021) is a scene-independent algorithm requiring only a query image, 3D model, and reference image with poses for camera relocalization. It uses metric learning for generalization across different scenes. KFNet (Zhou et al., 2020) combines a recursive network with Kalman filtering, extending SCR to the time domain for 2D to 3D correspondence. A system reported in a previous study (Brachmann E. and Rother C., 2021) estimated camera translation and orientation from RGB-D or RGB images. It was trained using a 3D environment model and required only RGB images and ground truth for training. Despite the strengths of SCR methods, their accuracy is limited by feature extraction, necessitating improvements in this regard.

Herein, the current research status of camera relocalization is reviewed. Existing deep learning–based camera relocalization methods can be categorized into three types: direct regression methods, image retrieval–based methods, and SCR methods. The specific principles of these methods are described as follows.

Direct regression methods: In these methods, the camera pose is directly estimated by inputting an image into a convolutional neural network and performing supervised learning. Although this approach is simple and requires only one neural network, its accuracy is generally low.

Image retrieval–based methods: These methods initially involve the feature encoding of input images. Then, they can directly find an image in the database that is the most similar to the query image and estimate the camera pose by matching their features. Alternatively, they can estimate their relative poses and more accurately estimate the camera pose. These methods demonstrate good generalization and adapt well to large-scale scenes. However, finding the most similar image from the image database is time-consuming. Additionally, differences between database and query images often make the retrieval of the most similar image challenging. Similar to traditional camera relocalization methods that establish sparse point clouds, optimizing the image-retrieval step helps narrow down the search space, facilitating faster estimation of the camera pose. The algorithm efficiency can be enhanced to some extent using global descriptors as the retrieval criterion.

SCR: Unlike traditional camera relocalization methods that rely on feature matching to establish the 2D–3D relation, SCR is more direct. By training a neural network, inputting an image, and obtaining the 3D positions of image pixels using the network, i.e., scene coordinates, the camera pose is calculated using the PnP-RANSAC algorithm based on the correspondence between 2D pixel points and 3D spatial coordinates. These methods considerably simplify the establishment of the 2D–3D relation. Unlike traditional camera relocalization and image retrieval–based methods, SCR does not directly store scene information in a database or 3D model. Instead, it implicitly expresses scene information using a neural network. A convolutional neural network is first trained to map 2D pixels to 3D spatial coordinates. Then, the spatial coordinates are input to the PnP-RANSAC algorithm for pose estimation. However, camera relocalization methods based on SCR may not perform as robustly in large-scale scenes as in small-scale scenes. In outdoor environments, the accuracy of camera relocalization may also be slightly influenced.

During camera relocalization in outdoor scenes, features extracted by an SCR network contain a large number of invalid scene coordinates, which can slightly affect the relocalization accuracy.To address this issue, herein, a voting segmentation network is adopted as the baseline model. However, using ResNet101 in the encoder of the voting segmentation network increases the model size and computational complexity; therefore, its immunity to external interference requires enhancement. To mitigate the impact of external noise on the encoder, pyramid convolution and 1 × 1 convolution kernels are used for constructing the main encoder. This design reduces the size of the network model and effectively enhances the feature extraction capability. Coordinate attention is also introduced to improve the resistance of the model to interference. Additionally, a more efficient feature extraction backbone network is developed using pyramid convolution to address object occlusion in outdoor scenes. Due to low texture and repetitive structures present in outdoor environments, the camera perceives the same scene from different poses, which in turn decreases the accuracy of camera relocalization. Furthermore, the spatial scene information of local features gradually diminishes as features propagate forward in the network. Thus, the output scene coordinates of a network do not provide usable scene information for estimating camera poses. To address these two issues, deep over-parameterized convolution modules are introduced into seg and vote decoders to improve the quality of scene image features while maintaining the original computational complexity.

This paper makes the following significant contributions.

(1) We propose a new network architecture, Py-layer, which is an encoding unit composed of stacked pyramid convolutions and 1 × 1 convolutions, with the addition of coordinate attention for feature correction. This simple and efficient network structure can extract multi-scale scene information, capture important features within the scene, and balance performance and efficiency.
(2) We use Py-layer to develop a camera relocalization solution based on a voting segmentation architecture. Deep over-parameterized convolution modules are integrated into the segmentation and voting decoders to address feature loss caused by repetitive structures and low-texture images in the scene.
(3) We conducted extensive experiments on the Cambridge Landmarks dataset to verify the effectiveness of our method in large-scale outdoor scenes. The experimental results show that, compared to VS-Net, which also uses a voting segmentation structure, our model improves average distance and angular accuracy by 29.41% and 33.33%, respectively, while reducing the number of parameters by 31.85%.

The remainder of this paper is organized as follows: Section 2 overviews deep learning–based camera relocalization methods. Section 3 provides the details of the proposed Py-Net network and its components. Section 4 presents the experimental results, and Section 5 summarizes the study with concluding remarks.

## 2 Materials and methods

Herein, the proposed methodology as well as the hardware and software platforms and datasets required for experiments are discussed.

### 2.1 Network architecture

**FIGURE 1:**
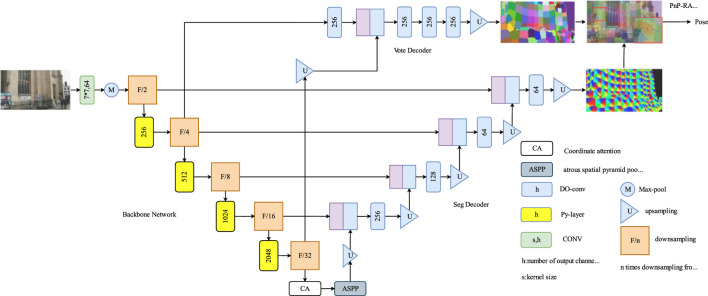
Py-Net network architecture. Py-Net architecture illustrates a encoder-decoder network, integrating an image encoder, segmentation decoder, and voting decoder, enabling accurate 2D-to-3D correspondence modeling.

The Py-Net architecture is a simple yet powerful encoder–decoder network (Figure 1) comprising three main components: the image encoder, seg decoder, and vote decoder. This design enables the generation of accurate landmark segmentation maps and voting maps for 2D-to-3D correspondence modeling. During training, Py-Net uses a landmark segmentation method based on patch labeling to generate segmentation coordinates for all pixels that correspond to patches surrounding the landmarks. Additionally, each pixel within the landmark patch predicts a 2D directional vector pointing toward the landmark, thereby enabling reliable and precise coordinate voting.

The main encoder comprises stacked Py-layers of different sizes. The Py-layer is a coding layer composed of 1 × 1 and pyramid convolutions. 1 × 1 convolution kernels perform dimensionality reduction and expansion, whereas pyramid convolutions use convolutions of multiple sizes to process the input image. It contains multiple levels of feature extraction layers, each with convolutions of different sizes and depths. Thus, the ability of the network to extract scene information enhances. Coordinate attention is added at the end of the Py-layer for feature correction. The output of the encoder is then fed into atrous spatial pyramid pooling, where features are sampled in parallel using dilated convolutions with different sampling rates. Finally, seg decoder and vote decoder branches produce landmark segmentation and voting maps, respectively.

In both decoder branches, depth over-parameterized convolution (DOConv) module was used as the convolution module. This design over-parameterizes the decoder, increasing the number of learnable parameters while maintaining the original computational complexity and thus enhancing the quality of scene image features.

### 2.2 Pyramidal convolution layer

Compared with standard convolution, pyramidal convolution offers a more robust capability to process input images. It uses multiple convolution sizes containing multiple levels of feature extraction layers, each with convolutions of different sizes and depths; thus, it captures rich details of the scene. Standard convolution contains only one convolution size, and the convolutional depth us equal to the depth of the feature map. In contrast, the convolution size increases and depth decreases in pyramidal convolution with increasing feature extraction levels. In outdoor scenes, the occlusion of building parts may occur, making it difficult for a single type of convolution to effectively capture details in the scene image. However, pyramidal convolution can use convolution kernels with different receptive fields to capture fine features, thereby improving the camera relocalization accuracy.

**FIGURE 2:**
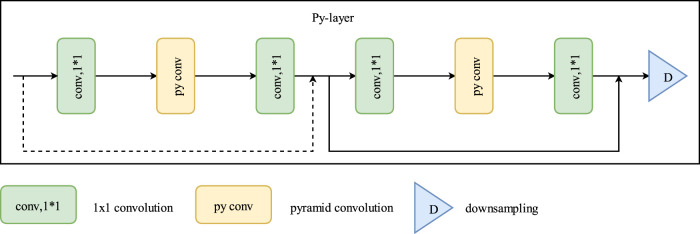
Py-layer network architecture. here pyramidal convolution increases the number of residual blocks. This allows the network to handle images with multiple convolution sizes and feature extraction levels, enhancing scene information extraction without increasing computational complexity.

Figure 2 shows that the number of residual blocks in the backbone network increases via pyramidal convolution, enabling the network to process images using multiple convolution sizes and multiple feature extraction levels. Thus, the ability of the network to extract scene information is enhanced. When optimizing the encoder of the voting segmentation network, the encoding function is mainly completed by 3 × 3 convolution kernels because 1 × 1 convolution kernels in each module serve to reduce and increase the dimensionality. Therefore, 3 × 3 convolutions are first improved in each module of the backbone encoder. Using pyramidal convolution, the network model can process input images using multiple sizes of convolution containing multiple levels of feature extraction layers, each with convolutions of different sizes and depths. Thus, the ability of the network model to extract scene information is slightly enhanced without introducing additional parameters and computational overhead.

For the input feature map ${FM}_{i}\in R^{C_{i}\times H\times W}$ , the n-layer pyramidal convolution has n convolutional kernel sizes $\left\{K_{1}^{2},K_{2}^{2},K_{3}^{2},\ldots ,K_{n}^{2}\right\}$ and n convolutional kernel depths $\left\{{FM}_{i},\frac{{FM}_{i}}{\left(\frac{K_{2}^{2}}{K_{1}^{2}}\right)},\frac{{FM}_{i}}{\left(\frac{K_{3}^{2}}{K_{1}^{2}}\right)},\ldots ,\frac{{FM}_{i}}{\left(\frac{K_{n}^{2}}{K_{1}^{2}}\right)}\right\}$ ; Output Features The ${FM}_{o}\in R^{C_{o}\times H\times W}$ is stitched together from the outputs of n convolutional kernels, where the size of $C_{o}$ is given by Equation 1:

$$C_{o}={FM}_{o1}+{FM}_{o2}+{FM}_{o3}+\ldots {FM}_{on}$$ (1)

Here ${FM}_{oi},i\in 1\ldots n$ is the output feature dimension corresponding to n convolutional kernels.

The number of parameters of the pyramidal convolution is given by Equation 2:

$$parameters=K_{n}^{2}∙\frac{{FM}_{i}}{\left(\frac{K_{n}^{2}}{K_{1}^{2}}\right)}∙{FM}_{0n}+K_{3}^{2}∙\frac{{FM}_{i}}{\left(\frac{K_{3}^{2}}{K_{1}^{2}}\right)}∙{FM}_{03}+K_{2}^{2}∙\frac{{FM}_{i}}{\left(\frac{K_{2}^{2}}{K_{1}^{2}}\right)}∙{FM}_{02}+K_{1}^{2}∙{FM}_{i}∙{FM}_{01}$$ (2)

Therefore, if the number of output feature maps is equal for each layer of the pyramidal convolution, then its parameters and computational costs will be evenly distributed across each level of the pyramid.

**FIGURE 3:**
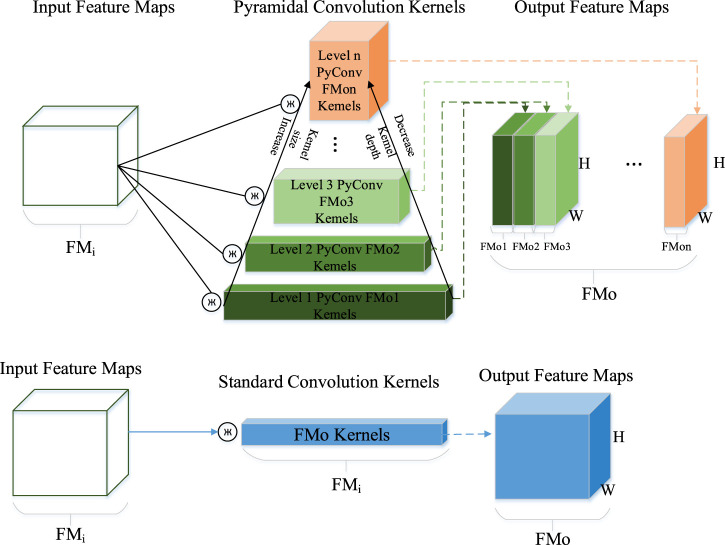
Comparative Analysis of pyramidal convolution and standard convolution in multi-level feature extraction and parameter count.

As shown in Figure 3, standard convolution can only have a single size convolution kernel, with the same kernel depth as the input features. Moreover, the number of computational parameters generated by a single convolution kernel is given by Equation 3:

$$parameters=K_{1}^{2}∙{FM}_{i}∙{FM}_{01}.$$ (3)

When multiple standard convolution kernels of different sizes are used to process input features, the as-generated computational and parameter quantities are greater than those generated via pyramidal convolution. This is because the input channels of these kernels must match the input features.

### 2.3 Coordinate attention

Attention mechanism is widely used in computer vision to enable network models to focus on relevant information. Some operations in convolutional neural networks, such as convolution, pooling, and fully connected layers, only consider nonself-desired clues. Contrarily, attention mechanism is purposeful and can explicitly model cues that align with its own intentions. As convolutions are conducted within local windows in the main encoder, their representation capability for global features is relatively weak. To address the issue of weak antiinterference capability in outdoor scenes with SCR networks, an attention mechanism is embedded in the encoder. This enables the network model to completely leverage global and local information. Consequently, the model can prioritize the areas of interest in an image, increase the corresponding weights of those areas, and ultimately highlight useful features while suppressing or ignoring irrelevant ones. These factors further enhance the localization accuracy.

**FIGURE 4:**
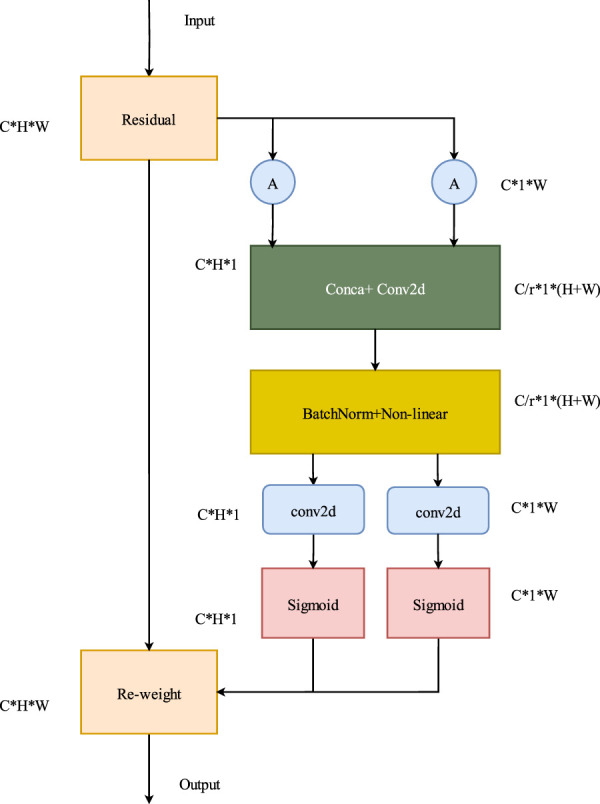
Visualization and Analysis of the coordinate attention mechanism.

Coordinate attention is a lightweight attention mechanism that considers channels and spaces in parallel (Figure 4). With the input of a feature X, X ∈ $R^{H\times W\times C}$ , the coordinated attention mechanism first pools along the height and width of the feature using two pooling kernels of size H × 1 and 1 × W, respectively. The output of height h and channel c can be expressed as follows (Equation 4):

$$z_{c}^{h}\left(h\right)=\frac{1}{W}\sum_{0\leq i\leq W}x_{c}\left(h,i\right)$$ (4)

The output of width w and the *c*th channel can be expressed as Equation 5:

$$z_{c}^{w}\left(w\right)=\frac{1}{H}\sum_{0\leq j\leq H}x_{c}\left(j,w\right)$$ (5)

where $z_{c}^{h}$ ∈ $R^{C\times H\times 1}$ and $z_{c}^{w}$ ∈ $R^{C\times 1\times W}$ .

After the two pooling operations, the two embedding features $z_{c}^{h}$ and $z_{c}^{w}$ can be stitched together. Then, the stitched features are reduced by 1 × 1 convolution and input to the sigmoid function (Equation 6):

$$f=\delta \left(F_{1}\left(z^{h},z^{w}\right)\right)$$ (6)

where $f\in R^{\frac{C}{r}\times 1\times \left(H+W\right)}$ , $F_{1}$ denotes the convolution, and δ denotes the sigmoid activation function. Then, the feature f is split in the spatial dimension using two 1 × 1 convolutions to obtain two feature maps. After transforming these maps, the attention vector is obtained as follows (Equations 7–8):

$$g^{h}=\sigma \left(F_{h}\left(f^{h}\right)\right)$$ (7)

$$g^{w}=\sigma \left(F_{w}\left(f^{w}\right)\right)$$ (8)

where ${\;F}_{w}$ and $F_{h}$ denote the 1 × 1 convolutional transformation and $f^{w}$ and $f^{h}$ denote the split features $f^{w}\in R^{\frac{C}{r\times w}}$ and $f^{h}\in R^{\frac{C}{r\times H}}$ , respectively. Eventually, the input features can be corrected using the two attention vectors (Equation 9):

$$y_{c}\left(i,j\right)=x_{c}\left(i,j\right)\times g_{c}^{h}\left(i\right)\times g_{c}^{w}\left(j\right)$$ (9)

CA has higher computational efficiency and less computational overhead in the spatial dimension than other attention modules.

### 2.4 Depthwise over-parameterized convolution

In standard convolution, all kernels in a convolutional layer are convolved with the input image. Standard convolution emphasizes the spatial relation of pixels and considers it as channels but at the expense of relatively high computational complexity. In contrast, depthwise convolution assigns each kernel to a specific input channel. Each channel of the input image is convolved with a dedicated kernel. Depthwise convolution focuses more on the features represented in the depth of an image and has relatively lower computational complexity.

Deep over-parameterized convolution (Cao et al., 2022) is an extension of standard convolution, in which an additional depthwise convolution is incorporated. This additional convolutional structure increases the number of trainable model parameters and enhances the learning capacity of the model. The quality of scene features extracted by this model is also consequently improved.

**FIGURE 5:**
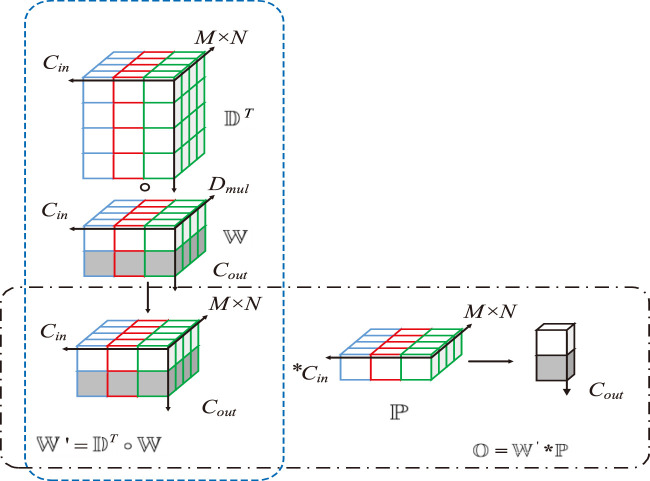
Depthwise over-parameterized convolution. By increasing the number of trainable model parameters, the learning capability of the model is enhanced. As a result, the quality of the extracted scene features is also improved.

The feature quality in the SCR network deteriorates due to the influence of repetitive structures and low-texture objects in scene images. To address this issue, standard convolutions must be substituted with deep over-parameterized convolutions. This replacement will enhance the quality of features extracted by the network and improve the overall nonlinear capability of the network, making it more robust. Figure 5 shows the structure of deep over-parameterized convolution.

Deep over-parameterized convolution comprises standard and depthwise convolutions. First, the convolutional kernel D from the standard convolution and the depthwise convolution kernel W are used to compute a new convolutional kernel W . Then, W is convolved with the feature P in the same manner as the standard convolutional kernel (Equation 10):

$$O=\left(D^{T}○W\right)\times p$$ (10)

**FIGURE 6:**
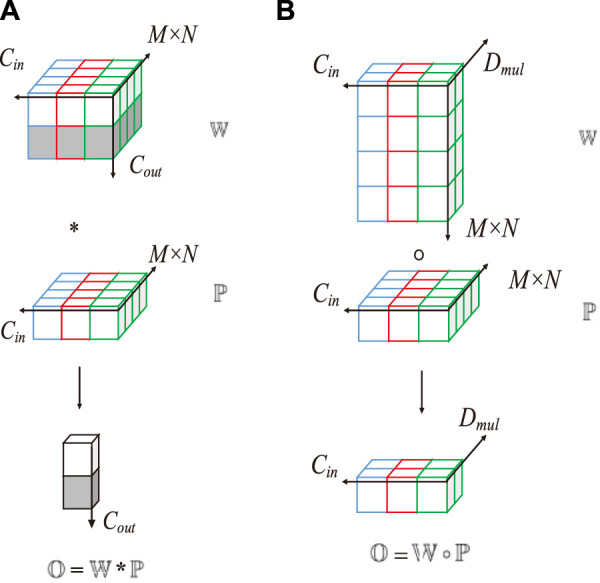
Computation methods of depthwise convolution and standard convolution. **(A)** Standard convolution, **(B)** DOConv.

As shown in Figure 6, W represents the convolutional kernels of standard and depthwise convolutions and P represents the feature. W in standard convolution is a 3D tensor, $w\in R^{c_{out}\times \left(M\times N\right)\times C_{in}}$ . P is a 2D tensor, $p\in R^{\left(M\times N\right)\times C_{in}}$ . where $C_{out}$ and $C_{in}$ denote the output and input channel dimensions of the feature and M×N denotes the spatial dimensions of the feature. Each convolutional kernel on the Cout dimension of W performs a dot product operation with P , yielding the output O (Equation 11):

$$O_{C_{out}}=\sum_{i}^{\left(M\times N\right)\times C_{in}}w_{C_{{out}^{i}}}p_{i}.$$ (11)

The dimensions of O correspond to the output channel dimension of the convolutional kernel.

In depthwise convolution, each input channel of P undergoes a dot product operation with $D_{mul}$ channels of the convolutional kernel. The dimension of each input channel of P is transformed from M×N to $D_{mul}$ , where $D_{mul}$ is the depth multiplier. The final output O is obtained accordingly (Equation 12):

$$O_{d_{mul}C_{in}}=\sum_{i}^{M\times N}w_{{id}_{mul}c_{in}}p_{{ic}_{in}},$$ (12)

where the depthwise convolutional kernel W is a 3D tensor, $w\in R^{\left(M\times N\right)\times D_{mul}\times C_{in}}$ .

DOConv and standard convolution have the same receptive field. For a feature input P , the output feature dimensions obtained via DOConv and output feature dimensions processed via standard convolution are identical. The linear transformation of standard convolution can be parameterized using $C_{out}\times \left(M\times N\right)\times C_{in}$ Cout training weights. The linear transformation of DOConv can be parameterized using the training weights of two convolutional kernels. Only when $D_{mul}\geq M\times N$ , the newly combined convolutional kernel W ʹ can exhibit the same linear transformation as the standard convolution kernel W when formed via combination. DOConv introduces over-parameterization to the network, thereby increasing the number of learnable parameters while maintaining the original computational complexity and enhancing the feature quality of scene images.

### 2.5 Loss function

The scene is first reconstructed in 3D using Py-Net. The resulting 3D surface is divided into 3D image patches using the center point of each patch as a 3D landmark, $\left\{q_{1},\ldots ,q_{n}\right\}\in R^{3}$ . Segmentation maps S $\in Z^{H\times W}$ and voting maps d $\in R^{H\times W\times 2}$ are obtained by projecting 3D image patches and 3D landmarks onto 2D images. To generate a segmentation map, values are assigned to each pixel point $p_{i}\left(u_{i},v_{i}\right)$ by determining its projected 3D image patch coordinate on the 2D image. A value of 0 is assigned to the pixel point coordinate $p_{i}\left(u_{i},v_{i}\right)$ if the associated area is not covered by the projected 3D surface, indicating that they did not influence the positioning. The landmark voting map is generated by first projecting the landmark $q_{j}$ on a 3D surface to a 2D plane to obtain 2D coordinates (Equation 13):

$$I_{j}=P\left(q_{j},K,C\right)\in R^{2},$$ (13)

where **K** is the camera internal reference matrix and C is the camera pose parameter. Each pixel belonging to an image patch containing the coordinate $q_{j}$ is responsible for projecting a 2D direction vector $d_{i}\in R^{2}$ pointing to $q_{j}$ . By unitizing this coordinate wth $p_{i}$ , the unit vector is obtained (Equation 14):

$$d_{i}=\frac{I_{j}-p_{i}}{{\left\|I_{j}-p_{i}\right\|}_{2}}$$ (14)

This vector is used to represent the orientation of the landmarks on the 2D plane. Supervised training of the voting segmentation network is possible by using the obtained segmentation map and voting map as training truths. This in turn establishes the relationship from 2D to 3D, thus enabling camera relocation.

The Vote Segmentation Network uses a prototype-based ternary loss function and a negative sample mining strategy to supervise the training of the network. Training the network in this way requires maintaining and updating a set of learnable class prototype embeddings P. Where each class prototype embedding represents a class and $P_{j}$ represents the class prototype embedding of the *j*th class. Therefore, the pixel embeddings belonging to the *j*th category should be as close as possible to, and as far away as possible from, the class prototype embeddings of other categories.

Pixel embeddings can be obtained by voting on the segmentation branches of the segmentation network and the class of prototype embeddings P. Pixel embeddings can form a pixel-by-pixel embedding graph E. The prototype-based ternary loss function first first L2 normalizes each pixel embedding in the embedding graph E, and then minimizes the error between each pixel embedding $E_{i}$ and the prototype embedding $P_{i}$ (Equation 15):

$$L_{seg}=\sum_{all\;i}\max\;\left(0,m+sim\left(E_{i},P_{i^{-}}\right)-sim\left(E_{i},P_{i^{+}}\right)\right)$$ (15)

where

$$\text{sim}\left(a,b\right)=\frac{a^{T}b}{\left\|b\right\|∙\left\|b\right\|}$$

is used to measure the cosine similarity between the pixel embedding and prototype-like embedding. $P_{i^{+}}$ denotes the true value of prototype-like embedding corresponding to pixel i and $P_{i^{-}}$ denotes the true value of prototype-like embedding unrelated to pixel i . m denotes the boundary of the loss function.

The voting decoder of the voting segmentation network is then used to determine the landmark’s cast position in the 2D image.The voting decoder is used to determine the casting position of landmarks in a two-dimensional image. Inputting an image, the voting decoder outputs a voting map d. For each pixel i in the input, it generates a two-dimensional direction vector $d_{i}$ , which points to the two-dimensional position of the landmark. The voting decoder is supervised trained under the voting loss function Equation 16:

$$L_{vote}\left(i\right)=\sum_{all\;i}1\left(S_{i}\neq 0\right)\left\|{\hat{d}}_{i}-d_{i}\right\|$$ (16)

Where one denotes the indicator function and ${\hat{d}}_{i}$ and $d_{i}$ denote the direction vectors predicted for pixel i and their direction vector truth values, respectively. The overall loss function of VS-Net is represented as in Equation 17:

$$L_{overall}=L_{seg}\left(i\right)+\lambda L_{vote}\left(i\right)$$ (17)

where λ denotes the weight of voting loss.

## 3 Results

This section evaluates the performance of our method, Py-Net, on the Cambridge Landmark dataset. We compared our results with those of existing camera relocalization methods, and the experiments demonstrated that the proposed method achieved state-of-the-art accuracy. Finally, we conducted ablation studies to investigate the contributions of individual components.

### 3.1 Dataset

The improvement in the proposed model was validated using the Cambridge Landmarks (Kendall Alex et al., 2015) dataset containing five outdoor landmarks scenes: Great Court, Kings College, Old Hospital, Shop Facade, and St. Mary’s Church. The dataset was more complex than indoor scenes; therefore, it better demonstrated the robustness of the model, as exterior environments undergo drastic environmental changes. For instance, the outdoor camera moves faster than the indoor camera, which may result in blurry images.

### 3.2 Evaluation metrics

As the camera relocalization error in outdoor environments is significant than that in indoor settings, using the a 1-cm distance error and 1° angle error as well as 2-cm distance error and 2° angle error are the evaluation metrics is not ideal. Therefore, the median distance error within 5 cm and median angle error of 5° were used as the evaluation metrics instead. We also report the percentage of high-precision localization points with a distance error within 5 cm and an angle error within 5°

### 3.3 Results and analysis

The performance of Py-Net was compared with existing visual localization systems on the Cambridge Landmarks dataset. Detailed results are shown in Table 1, where the primary indicators for evaluating the accuracy of camera relocalization are compared: translation error (m) and rotation error (°). Experimental results indicate that Py-Net considerably outperforms the existing methods. The translation error decreased by 40% using the SCR method than DSAC. This indicates that the voting segmentation network can considerably reduce the number of outliers in the scene and improve the relocalization accuracy. Py-Net outperformed VS-Net, which also employs a voting segmentation architecture, in the majority of scenes, particularly in the Great Court scene, where the translation and rotation errors decreased by 14% and 50%, respectively. This suggests that using Py-layer to enhance the backbone encoder, the network can learn multiscale features. Thus, the ability of backbone encoder to extract scene information enhances, resulting in better performance.

**TABLE 1:** Visual localization accuracy of state-of-the-art methods.We report the median translation error (m), rotation error (degrees), and localization precision, where the translation and rotation errors are within 5 cm and 5°, respectively. NA indicates no available values, Best results are in bold.

		Great court	Kings college	Old hospital	Shop facade	St.Mary’s church	Avg
Direct Regression	PoseNet (Kendall et al., 2015b)	NA	1.92 m,5.40 °	2.31 m,5.38 °	1.46 m,8.08 °	2.65 m,8.48 °	2.09 m,6.84 °
	Dense PoseNet (Shen and Chen, 2019)	NA	1.66 m,4.86 °	2.57 m,5.14 °	1.41 m,7.18 °	2.45 m,7.96 °	2.02 m,6.29 °
	LSTM-Pose (Luo et al., 2018)	NA	0.99 m,3.65 °	1.51 m,4.29 °	1.18 m,7.44 °	1.52 m,6.68 °	1.30 m,5.52 °
	SVS-Pose (Abozeid et al., 2022)	NA	1.06 m,2.81 °	1.50 m,4.03 °	0.63 m,5.73 °	2.11 m,8.11 °	1.32 m,5.17 °
	NeRF-loc (Liu et al., 2023) Pixloc (Sarlin et al., 2021b) ST-Pixloc (Wang et al., 2024)	0.25 m,**0.1** °	**0.07** **m**,**0.2** °	0.18 m,0.4 °	0.11 m,**0.2** °	**0.04** **m**,**0.2** °	0.13 m,0.22 °
		0.42 m,0.18 °	0.16 m,0.26 °	0.49 m,0.79 °	0.06 m,0.23 °	0.14 m,0.36 °	0.25 m,0.36 °
		0.24 m,0.13 °	0.15 m,0.23 °	0.42 m,0.69 °	0.05 m,0.26 °	0.13 m,0.31 °	0.19 m,0.32 °
SCR	DSAC (Brachmann et al., 2017)	2.80 m,1.5 °	0.30 m,0.5 °	0.33 m,0.6 °	0.09 m,0.40 °	0.55 m,1.6 °	0.81 m,0.92 °
	DSAC++ (Brachmann and Rother, 2021b)	0.4 m,0.2 °	0.18 m,0.3 °	0.20 m,**0.3** °	0.06 m,0.3 °	0.13 m,0.4 °	0.19 m,0.3 °
	ACE (Brachmann et al., 2023)	0.42 m,0.2 °	0.28 m,0.4 °	0.31 m,0.6 °	**0.05** **m**,0.3 °	0.19 m,0.6 °	0.25 m,0.42 °
	VS-Net (Huang et al., 2021)	0.22 m,0.2 °	0.16 m,0.3 °	**0.16** **m**,0.4 °	0.06 m,**0.2** °	0.08 m,0.4 °	0.14 m,0.3 °
	Py-Net (**ours**)	**0.19** **m**,**0.1** °	0.14 m,**0.2** °	**0.16** **m**,**0.3** °	**0.05**,m**0.2** °	0.07 m,**0.2** °	**0.12** **m**, **0.2** °

**FIGURE 7:**
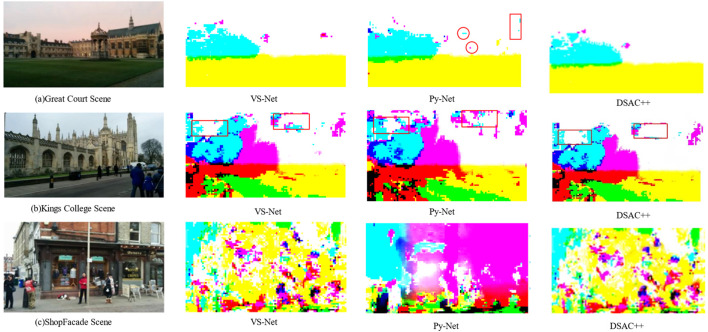
Comparison of scene coordinate predictions. **(A)** Great court snene; **(B)** Kings College Scene; **(C)** ShopFacade Scene. The network predicts the 2D-3D correspondences of the image, visualizing them as a scene coordinate map by rendering different coordinates in different colors. The richness of the scene information in the scene coordinate map significantly affects the accuracy of the PnP algorithm.

Figure 7 compares the scene coordinate prediction between VS-Net and Py-Net. In outdoor scenes, the enlarged scene scale results in a substantial number of invalid scene coordinate points. This affects the prediction accuracy and limits the scene information available for camera relocation.

However, as shown in Figure 7A, Py-Net provides higher amounts of usable information within the predicted scene coordinate maps. In the Great Court scene, Py-Net generates a scene coordinate map with an increased level of scene information (Figure 7A). In the Kings College scene, the scene coordinate map predicted by Py-Net contains noticeably increased usable information (Figure 7B). The Shop Facade scene contains a substantial amount of usable scene information, with fewer background pixels representing the sky (Figure 7C). This indicates that incorporating depthwise separable convolutional modules into the scene coordinate decoder effectively enriches the amount of scene information, thereby enhancing the information representation capability of the network.

**FIGURE 8:**
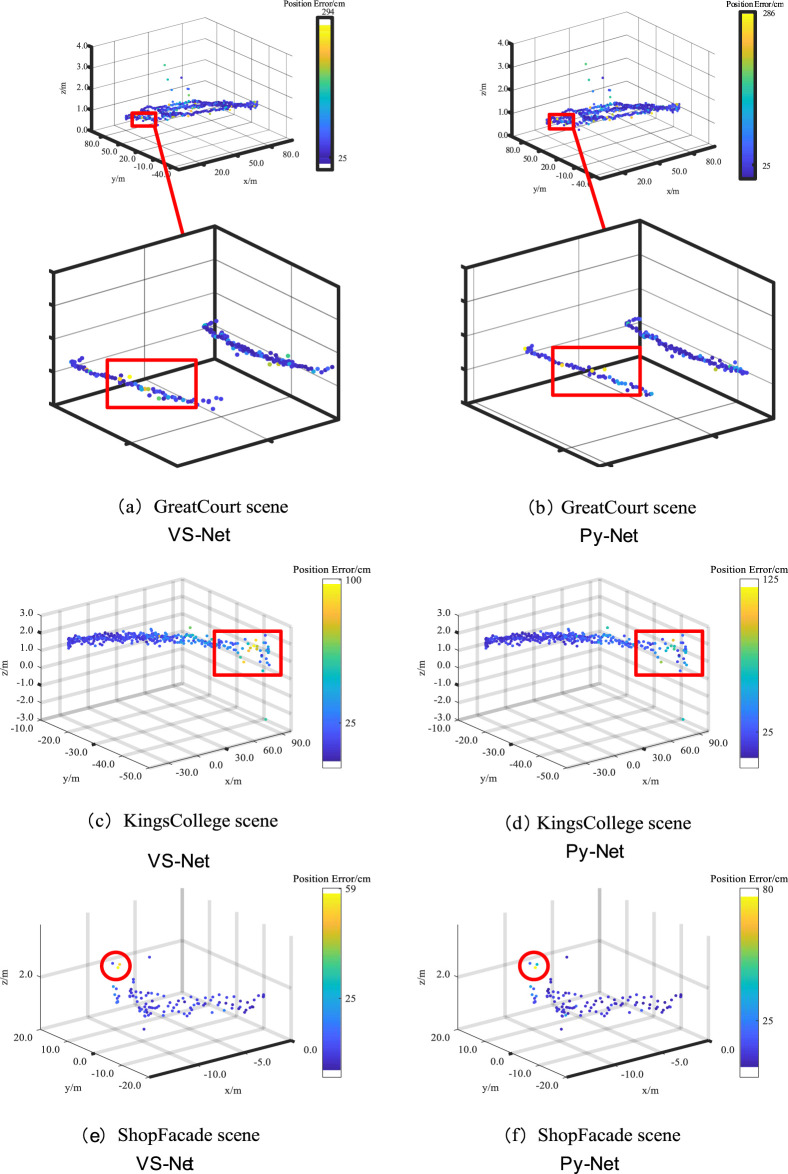
Comparison of Localization Trajectories on the Cambridge Landmark Dataset: VS-Net **(A, C, E)** vs. Improved Py-Net **(B, D, F)**.

Figure 8 shows the positioning trajectories of the improved Py-Net on the Cambridge Landmark dataset. The figure compares the distance errors between VS-Net and Py-Net. Panels (a), (c), and (e) show the positioning trajectories of VS-Net, and panels (b), (d), and (f) show the positioning trajectories of the improved Py-Net. When zooming in on the positioning errors in the Great Court scene, a certain degree of reduction in distance errors can be observed. In the Kings College scene, Py-Net reduces the distance errors of marked points. As the test samples of the Shop Facade scene were limited, only a few positioning points with reduced errors are shown in the figure.

**FIGURE 9:**
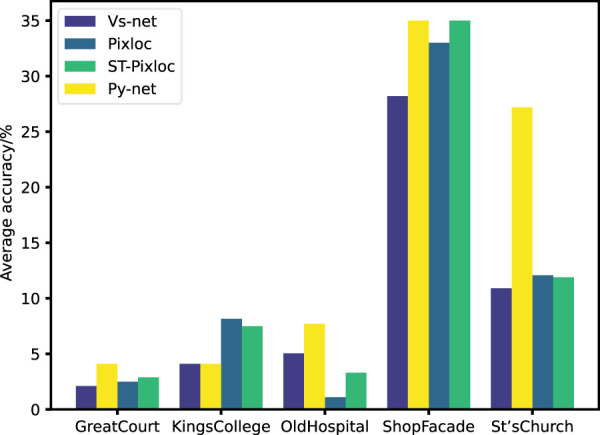
Percentage of localization points within 5 cm 5° on the Cambridge Landmark Dataset.

Figure 9 shows that in multiple scenes, Py-Net outperforms existing methods in the proportion of high-precision points with a distance error of less than 5 cm and an angle error of less than 5°. The increase in the proportion of high-precision localization points indicates that the number of invalid localization points in the scene has decreased, meaning that outliers have been filtered out.

The Py-layer and depthwise separable convolution were introduced to enhance the performances of the main encoder and decoder, respectively. As a result, the size of Py-Net considerably compared with that of VS-Net (Table 2). Specifically, the model size decreased from 236 to 170 MB, and the parameter count was only 68.15% of the original. This improvement considerably enhanced the applicability of the model on devices with limited storage space.

**TABLE 2:** Comparison of model sizes (MB).

	VS-Net	Py-Net
Model Size	236	170
Parameters	61,864,994	42,163,554

Ablation experiments were conducted to demonstrate the effectiveness of the improvements proposed herein. By introducing the Py-layer and coordinated attention mechanism, Py-Net can effectively extract outdoor scene information and filter out invalid localization points in the scene. Thus, the model performance is improved across various scenes. In the Great Court scene, the distance error decreased by 16%, whereas in the Shop Facade and St. Mary’s Church scenes, the angle error decreased by 33%. As shown in Table 3, after incorporating the depthwise separable convolution module, the angle error of the network considerably increased across multiple scenes. This indicates that Py-Net can retain more scene information, thus achieving more accurate estimation results.

**TABLE 3:** Ablation experiment√ indicates the component is used, × indicates the component is not used.

Components	“√” considering component “ⅹ” excluding component		
Py-layer + CA	ⅹ	ⅹ	√
DoConv	ⅹ	√	√
Great Court	0.22 m, 0.2°	0.22 m, 0.1°	0.19 m, 0.1°
Kings College	0.16 m, 0.3°	0.16 m, 0.2°	0.15 m, 0.2°
Old Hospital	0.16 m, 0.4°	0.16 m, 0.3°	0.16 m, 0.3°
Shop Facade	0.06 m, 0.2°	0.06 m, 0.3°	0.06 m, 0.2°
St. Mary’s Church	0.08 m, 0.4°	0.08 m, 0.3°	0.07 m, 0.2°
Average	0.14 m, 0.3°	0.14 m, 0.2°	0.13 m, 0.2°

## 4 Conclusion

This section summarizes the methods employed and the key findings of the study. The methods involved enhancing the performance of VS-Net for camera relocalization in outdoor scenes by optimizing its backbone network and improving feature extraction capability. The study resulted in a 14% increase in average translation accuracy on the Cambridge Landmark dataset, accompanied by a 30% reduction in model size.

Regarding potential real-time applicability, the optimized VS-Net model shows promise for real-time camera relocalization applications, particularly in outdoor environments. Future research directions may include further exploration of feature extraction techniques, investigating the model’s robustness in various environmental conditions, and integrating additional sensor modalities for improved performance and versatility.

## Data Availability

The datasets presented in this study can be found in online repositories. The names of the repository/repositories and accession number(s) can be found in the article/supplementary material.
